# Selected neonicotinoids and associated risk for aquatic organisms

**DOI:** 10.17221/78/2023-VETMED

**Published:** 2023-08-31

**Authors:** Alzbeta Strouhova, Josef Velisek, Alzbeta Stara

**Affiliations:** Laboratory of Aquatic Toxicology and Ichtyopathology, Faculty of Fisheries and Protection of Waters, Research Institute of Fish Culture and Hydrobiology, South Bohemian Research Center of Aquaculture and Biodiversity of Hydrocenoses, University of South Bohemia in České Budějovice, Vodňany, Czech Republic

**Keywords:** acetamiprid, aquatic ecosystems, flupyradifurone, nicotinic acetylcholine receptors agonists, thiacloprid, toxicity

## Abstract

Neonicotinoids are one of the newest groups of systemic pesticides, effective on a wide range of invertebrate pests. The success of neonicotinoids can be assessed according to the amount used, for example, in the Czech Republic, which now accounts for 1/3 of the insecticide market. The European Union (EU) has a relatively interesting attitude towards neonicotinoids. Three neonicotinoid substances (imidacloprid, clothianidin and thiamethoxam) were severely restricted in 2013. In 2019, imidacloprid and clothianidin were banned, while thiamethoxam and thiacloprid were banned in 2020. In 2022, another substance, sulfoxaflor, was banned. Therefore, only two neonicotinoid substances (acetamiprid and flupyradifurone) are approved for outdoor use in the EU. Neonicotinoids enter aquatic ecosystems in many ways. In European rivers, neonicotinoids usually occur in nanograms per litre. Due to the low toxicity of neonicotinoids to standard test species, they were not expected to significantly impact the aquatic ecosystem until later studies showed that aquatic invertebrates, especially insects, are much more sensitive to neonicotinoids. In addition to the lethal effects, many studies point to sublethal impacts - reduced reproductive capacity, initiation of downstream drift of organisms, reduced ability to eat, or a change in feeding strategies. Neonicotinoids can affect individuals, populations, and entire ecosystems.

## INTRODUCTION

Pesticides play an important role in ensuring a sustainable food supply all over the world. Their use can reduce the agricultural losses and also improve the affordability and quality of the food (Hedlund et al. 2019; Umetsu and Shirai 2020; Tudi et al. 2021). Pest management is part of agriculture since it started about 10 000 years ago. The development and use of pesticides can be divided into several stages, depending mainly on the origin of the pesticide substances. Until about the middle of the 19^th^ century, the used substances were mainly of natural origin, derived from plants, animals, or minerals. The second half of the 19^th^ century and the beginning of the 20^th^ century were associated with the use of inorganic substances or by-products of industrial production. During the Second World War and subsequently until about the 1970s, synthetically produced organic substances were widely used (Umetsu and Shirai 2020). The discovery of dichlorodiphenyltrichloroethane (DDT) and subsequent warnings about its negative effects can be considered a turning point. Therefore, since the 1970s, the emphasis has been placed on the development and use of synthetic organic pesticide substances with lower risk to humans and non-target organisms (Jarman and Ballschmiter 2012; Harada et al. 2016; Sharma et al. 2019; Umetsu and Shirai 2020).

Pesticides are widely used even though could potentially be a risk to the water quality, biodiversity, and also human health. About 64% of global agricultural land is at risk of pesticide pollution by more than one active ingredient of pesticides (Tang et al. 2021). In 2020, 2.7 million tonnes of active ingredients were globally applied, which represent 7.2 million tonnes of formulated products with a value of 41.1 billion USD. About 18% of those substances were insecticides. The major contributing countries in pesticide usage are the USA, followed by Brazil, China, Argentina, and the Russian Federation (FAO 2022). Generally, pesticides are categorised, according to the target organism, into herbicides, insecticides, fungicides, bactericides, rodenticides, etc. (Abubakar et al. 2019; Hassaan and El Nemr 2020). According to the Food and Agricultural Organization of the United Nations (FAO 2022), the most common insecticides that are used worldwide are chlorinated hydrocarbons, organophosphates, carbamates–insecticides and pyrethroids. One of the most rapaciously developing group of insecticides are nicotinic insecticides (Umetsu and Shirai 2020).

## NEONICOTINOIDS

These compounds are synthetically produced, originating from nicotine, and were launched on the market in the 1990s. Neonicotinoids are highly effective against a wide range of pests. They accounted for nearly 23% of the global insecticide market in 2016 (Morrissey et al. 2015; Casida 2018; Klingelhofer et al. 2022). The tobacco leaf extract was used to control garden plant pests as early as the end of the 17^th^ century.

The active ingredient in these extracts is the alkaloid – nicotine; however, pure nicotine was not isolated before 1828 (Cremlyn 1978). In the 1970s, there were attempts to increase the usage of nicotinoids – natural substances with a similar structure to nicotine. Still, these compounds were not very practical to use commercially for plant protection due to their ease of photo degradability. After studies on the structural activity and the replacement of some components, highly effective and, at the same time, a photostable analogy of natural nicotine-neonicotinoids were formed. The first one, nithiazine, was synthesised in 1977. Nithiazine was followed by other heterocyclic compounds – imidacloprid (1985), thiacloprid (1985) and thiamethoxam (1992). At the same time, acyclic compounds were produced – nitenpyram (1988), acetamiprid (1989), clothianidin (1989) and dinotefuran (1994). A significant difference between nicotinoids and neonicotinoids is the absence of the ionisable basic amine or imine substituent (Tomizawa and Casida 2005).

In 1991, imidacloprid was launched, becoming the best-selling insecticide worldwide. This success was followed by nitenpyram and acetamiprid in 1995 and thiamethoxam in 1998. After 2000, three other compounds were launched on the market – thiacloprid (2000), clothianidin (2001), and dinotefuran (2002) (Bass et al. 2015). All those compounds are called “second-generation neonicotinoids”. Nicotine and the other compounds synthesised before imidacloprid are considered the first-generation. Nicotinic insecticides developed or launched after 2010, such as sulfoxaflor, flupyradifurone, flupyrimin, triflumezopyrim or dicloromezotiaz are considered third-generation neonicotinoids (Umetsu and Shirai 2020). The Insecticide Resistance Action Committee (IRAC) classifies nicotinic insecticides as Group 4 – Nicotinic acetylcholine receptor agonists. Group 4 includes nicotine, neonicotinoids, sulfoximines, butenolides, mesoionics and pyridylidenes (IRAC 2023). A detailed classification of the different nicotinic insecticides is shown in Table 1. All the insecticides from this group principally share the same binding site on the nicotinic acetylcholine receptors (NAChRs) and are therefore considered as sharing the same mode of action. The sub-classification is based on structural differences in the insecticide molecules (IRAC 2015). However, the Pesticide Action Network Europe (PAN Europe 2016) counters that, although the structures of flupyradifurone and sulfoxaflor are different, they are still neonicotinoid insecticides. For this reason, flupyradifurone should be treated accordingly by the regulator, considering its systemic nature and the harm it could cause to non-target organisms.

**Table 1:** Classification of nicotinic acetylcholine receptor agonists (IRAC 2023)

Group 4 – Nicotinic acetylcholine receptors agonists					
	neonicotinoids	sulfoximines	butenolides	mesoionics	pyridylidenes
Nicotine	acetamiprid (ACE) clothianidin (CLO) dinotefuran (DNT) imidacloprid (IMI) nitenpyram (NTP) thiacloprid (THA) thiamethoxam (THM)	sulfoxaflor (SFX)	flupyradifurone (FLU)	triflumezopyrim dicloromezotiaz	flupyrimin

### Mechanism of the toxic effect of neonicotinoids

Neonicotinoids are classified as systemic insecticides and as neurotoxins acting on the central nervous system of organisms (Wang et al. 2018a). They work in insects and mammals as nicotinic acetylcholine receptor (nAChRs) agonists, especially the subtype α4β2 (Tomizawa and Casida 2005).

Acetylcholine (ACh) is an endogenous agonist and excitatory neurotransmitter of the cholinergic nervous system. It occurs under the action of a nicotinic cholinergic synapse in two steps. Acetylcholine is first released through the presynaptic membrane and interacts with a localised binding site on the extracellular domain nAChR complex-ion channel. A conformational change in the receptor molecule leads to the opening of the ion channel, promoting the influx of extracellular Na^+^ and intracellular K^+^, disturbing the equilibrium membrane potential. In insects, most nAChRs are located in the neutrophilic areas of the central nervous system. They are responsible for fast neurotransmission and are an important target for insecticides (Tomizawa and Casida 2005). Mammals have nAChRs mainly in the muscles, brain, and peripheral vegetative nerves. They work as chemically dependent ion channels, composed of five subunits forming vertical pores in the plasma membrane of cells (Yamamoto et al. 1998).

Vertebrates and invertebrates have different nAChRs, so neonicotinoids are thought to have a higher selectivity for invertebrate nAChRs than vertebrates. This phenomenon is the reason for the lower neurotoxicity of neonicotinoids for mammals, fish, and birds. Vertebrate receptors have a different configuration in the receptor-forming subunits, and insecticide binding is weaker or takes less time than it does with the insects (Yamamoto et al. 1998; Tomizawa and Casida 2005).

Neurotoxicity is not the only possible toxic effect of neonicotinoids (Casida 2011; Casida 2018; Thompson et al. 2020; Mukherjee et al. 2022). Studies indicate that, for vertebrates and also invertebrates, they may be genotoxic (Hong et al. 2018; Senyildiz et al. 2018), immunotoxic (Di Prisco et al. 2017; Hong et al. 2018), hepatotoxic (Wang et al. 2019), and have cytotoxic effects (Senyildiz et al. 2018; Wang et al. 2019). Some studies also (Bal et al. 2012; Lonare et al. 2014; Wessler and Kirkpatrick 2017; Ge et al. 2018; Raby et al. 2018; Picone et al. 2022) point to the possible impairment to the reproductive processes and abilities of vertebrate and invertebrate animals when exposed to neonicotinoid substances.

### European Union and neonicotinoids

In the mid-1990s, shortly after the first neonicotinoids’ launch, French beekeepers warned of the loss of bees caused by the newly introduced class of systemic insecticides, particularly by the compound imidacloprid. Beekeepers reported extensive damage to foraging hives on the crops treated with imidacloprid. However, poisoning symptoms indicated more of the parasitic mite Varroa and its associated viruses (Ndakidemi et al. 2016). At the European Conference on Bee Research in 2006, Italian scientists warned of the dangers of sowing dust treated with clothianidin and imidacloprid (Greatti et al. 2006). The risk of the dust from the infested seeds was confirmed by a massive bee poisoning incident in southern Bavaria in the Rhine Valley. More than 11 500 hives showed signs of insecticide poisoning. A chemical analysis of the dust, plant samples, bee samples and pollen confirmed the poisoning was derived from clothianidin treated corn seeds (Pistorius et al. 2008). Four major studies were published in 2012 (Gill et al. 2012; Henry et al. 2012; Lu et al. 2012; Whitehorn et al. 2012), suggesting that neonicotinoids are dangerous for bees. Even though the studies contained shortcomings in the form of unrealistically simulated laboratory conditions or excessive doses of the administered pesticide, the studies made a significant contribution to the European Commission’s decision on a moratorium on the use of three neonicotinoids (imidacloprid, clothianidin and thiamethoxam) on crops attractive to bees from December 2013. The moratorium was based on laboratory studies that do not match the natural environment and bee behaviour, confusing, especially for beekeepers, who have long moved bee colonies close to flowering oilseed rape (*Brassica napus* subsp. *napus*), from whose nectar they can obtain prised honey. The moratorium is also problematic for farmers who use funds to replace the prohibited substances thus causing financial difficulties (Carreck 2017). In 2013, with Regulation No. 485/2013, the European Commission severely limited the use of plant protection products and seed treatments containing clothianidin, imidacloprid, or thiamethoxam. Measures based on a risk assessment by the European Data Protection Supervisor Food Safety Authority (EFSA) in 2013 were concerned with bee-attractive plants, such as maize, oilseed rape or sunflowers. Using pesticides containing the three substances was only possible in greenhouses, treating certain crops after flowering, or treating winter cereals. In 2017, the competent services of the European Commission submitted a proposal for a total ban on the use of these three active substances in the outdoor environment. Implementing a regulation amending the conditions for the approval of the active substances imidacloprid, clothianidin, and thiamethoxam were published in the Official Journal of the EU on 30 May 2018. The use of all three substances in the outdoor environment is prohibited and remains valid as only possible in permanent greenhouses. Other neonicotinoid substances were also evaluated – acetamiprid and thiacloprid. Acetamiprid is considered as having low toxicity to bees, and its use is approved in the EU until 28 February 2033. National authorities can assess whether there are more favourable alternatives to the used product, including non-chemical methods. The use of clothianidin and imidacloprid was definitively restricted in 2019 and thiamethoxam and thiacloprid were restricted in 2020. From 2020, some European Member States have repeatedly granted emergency authorisations for the mentioned banned substances for their use on sugar beets, but the European Commission and EFSA are analysing and monitoring these steps and discussing possible wider implications of the ruling (European Commission 2023). Only 7 years after its authorisation, the use of sulfoxaflor was restricted by a European Commission decision in April 2022. Member States withdrew or amended authorisations for plant protection products containing sulfoxaflor as an active substance by 19 November 2022 at the latest [Reg. (EU) 2022/686]. Therefore, only acetamiprid and flupyradifurone are approved for use in the EU. Although most active substances from the group of neonicotinoids are banned in the EU, these substances, mainly imidacloprid and thiacloprid, are still among the most widely used insecticides in the world, especially in China and the USA (Klingelhofer et al. 2022).

### Neonicotinoids in aquatic ecosystems

Neonicotinoids are soluble in water, making them easier to use, such as a systemic insecticide. They also have different half-lives in the soil and water, where they are under anaerobic conditions and at neutral or slightly acidic pH resistant to hydrolysis (EFSA 2008; Morrissey et al. 2015). Persistence is affected by environmental conditions, such as an increased pH, and the turbidity increases the persistence (Sarkar et al. 2001). Neonicotinoids may be subject to shallow water with high transparency photodegradation. Physical-chemical properties, especially high solubility, and low soil adsorption support the movement of these pesticides through the surface and subsurface runoff (EFSA 2008).

Neonicotinoids enter aquatic ecosystems mainly through surface runoff from treated cultures (Armbrust and Peeler 2002) by leaching into the groundwater (Kreutzweiser et al. 2008), by treating cultures and sowing infested seeds in water formations, such as in rice fields (Lamers et al. 2011). During the sowing of seeds treated with neonicotinoid preparations, dust is formed, obtained as a solid fraction into the recipients in the form of fallout (Morrissey et al. 2015). Significant contamination of the surface water occurs after heavy precipitation (Chiovarou and Siewicki 2008) and during snow melts, which can carry both dissolved and solid fractions (Main et al. 2014).

Neonicotinoids have become relatively commonly detected substances in aquatic ecosystems worldwide. In surface waters, they are generally detected in the tens to hundreds of ng/l, with exceptional concentrations in the tens of μg/l (Main et al. 2014 Morrissey et al. 2015; Pietrzak et al. 2019; Sjerps et al. 2019; Lu et al. 2020; Mahai et al. 2021). The limiting concentrations for the occurrence of pesticides in drinking water in the EU are set by Directive (EU) 2020/2184 at 0.1 μg/l for each individual pesticide or its metabolite and at 0.5 μg/l for the sum of the individual pesticide concentrations set (European Commission 2020). An overview of the detected concentrations of neonicotinoids in water is given in Table 2. In general, the most widespread neonicotinoid in surface waters is imidacloprid, with data also available for acetamiprid, thiacloprid, possibly clothianidin, and thiamethoxam. The concentrations and abundance in surface waters may be influenced, to some extent, by using the surrounding landscape or the time of year when the sampling is carried out. Higher concentrations of neonicotinoids can be expected in agricultural areas and during periods of insecticide application. Neonicotinoids are also detected in sources of drinking water. If a chemical is present in the water or its residue, aquatic organisms have a minimal possibility to escape. The way how the substance affects the organism depends on its concentration, kinetics, mechanism of action and the detoxification ability of the species (Escher et al. 2011). Pesticides can enter the bodies of organisms, for example, by inhalation, together with food or passage through the epidermis (Pisa et al. 2015).

**Table 2:** Concentrations of neonicotinoids in global waters

Study location		Type of water	Neonicotinoid concentration (ng/l)						References
Country	location		year	acetamiprid	thiacloprid	clothianidin	imidacloprid	thiamethoxam	
Czech Republic	Úhlava River	DWTP (raw water)	–	–	–	–	11.53	–	Troger et al. (2021)
	mean from 18 surface water sampling locations		2014	5	5.82	–	–	–	CHMI (2023)*
	mean from 13 surface water sampling locations		2015	5	5	–	–	–	
	mean from 137 surface water sampling locations		2016	6.74	7.63	–	–	–	
	mean from 238 surface water sampling locations		2017	6.77	7.25	–	–	–	
	mean from 261 (acetamiprid) and 368 (thiacloprid) surface water sampling locations		2018	6.35	6.9	–	–	–	
	mean from 246 (acetamiprid) and 350 (thiacloprid) surface water sampling locations		2019	5	5.35	–	–	–	
	mean from 223 (acetamiprid) and 304 (thiacloprid) surface water sampling locations		2020	5.1	4.75	–	–	–	
	mean from 184 (acetamiprid) and 273 (thiacloprid) surface water sampling locations		2021	5.07	4.77	–	–	–	
	mean from 251 (acetamiprid) and 347 (thiacloprid) surface water sampling locations		2022	5.47	4.39	–	–	–	
Austria	Schwarzau	river water	2018	–	0.7	12	< LOD (2.5 ng/l)	–	Casado et al. (2019)
	Stiefing	river water	2018	< LOD (5 ng/l)	< LOD (0.5 ng/l)	10.7	< LOD (2.5 ng/l)	< LOD (2.5 ng/l)	
Belgium	Moubeek	canal water	2018	–	–	–	3.4	–	Casado et al. (2019)
	Wulfdambeek	canal water	2018	< LOD (5 ng/l)	–	–	4.3	< LOD (2.5 ng/l)	
	De Wamp	canal water	2018	–	21.5	–	6	< LOD (2.5 ng/l)	
Denmark	Hove	river water	2018	–	–	–	25.7	–	Casado et al. (2019)
	Skensved	river water	2018	–	–	20.9	–	< LOD (2.5 ng/l)	
France	Ruisseau de la Madoire	river water	2018	–	–	–	5.1	–	Casado et al. (2019)
	Le Gouessant	river water	2018	–	2.9	–	6.3	–	
Germany	Ems	river water	2018	–	< LOD (0.5 ng/l)	–	34.5	–	Casado et al. (2019)
	Essener	canal water	2018	–	< LOD (0.5 ng/l)	–	2.6	–	
	Soeste	river water	2018	–	< LOD (0.5 ng/l)	–	8.5	10.1	
Italy	Lake	DWTP (raw water)	–	2.09	–	–	1.98	–	Troger et al. (2021)
	Mariana Mantovana	canal water	2018	–	–	< LOD (5 ng/l)	5.1	2.5	
	Roggia Saverona	river water	2018	–	–	< LOD (5 ng/l)	5.8	9.4	Casado et al. (2019)
	Cumigano sul Nauiglio	canal water	2018	–	–	–	< LOD (2.5 ng/l)	2.5	
Poland	Wkra	river water	2018	–	–	–	7.5	–	Casado et al. (2019)
	Mlawka	river water	2018	< LOD (5 ng/l)	–	–	5.9	–	
Portugal	Alquera Reservoir	surface water	2017–2018	–	5.7	–	–	7.9	Palma et al. (2021)
	Guadiana Streams	surface water	2017–2018	–	5.6	–	60.8	8.6	
Spain	Tagus River	surface water	2020	0.05–3.55	0.04–1.43	0.04–2.54	0.28–10.18	0.04–2.39	Casillas et al. (2022)
	Turia River	surface water	2012	–	–	–	8.04	–	Ccanccapa et al. (2016)
	Turia River	–	2013	–	–	–	3.54	–	
	Llobregat River	surface water	2016	8–15	–	–	5–447	–	Quintana et al. (2019)
	Llobregat River	–	2017	6–14	–	–	5–215	–	
	Llobregat River	ground water	2016	–	–	–	5–16	–	
	Llobregat River	ground water	2017	–	–	–	5–10	–	
	Besós River	ground water	2016	–	–	–	23–25	–	
	Besós River	ground water	2017	–	–	–	7–27	–	
	Barcelona	DWTP (raw water)	2016	–	–	–	5–6	–	
	Barcelona	DWTP (raw water)	2017	7	–	–	5–51	–	
	Rioja Baja	surface water	2019	–	–	–	4–70	–	Manjarres-Lopez et al. (2021)
	not specified	DWTP (raw water)	–	8.1	–	–	19.86	–	Troger et al. (2021)
	Flúmen	river water	2018	–	1.3	–	9.4	–	Casado et al. (2019)
	Segre	river water	2018	< LOD (5 ng/l)	3.7	< LOD (5 ng/l)	47.1	< LOD (2.5 ng/l)	
United Kingdom	Otter	river water	2018	–	< LOD (0.5 ng/l)	–	13.9	–	Casado et al. (2019)
	Tale	river water	2018	–	< LOD (0.5 ng/l)	< LOD (5 ng/l)	7.2	–	
Argentina	Tapalqué River	surface water	2014–2015	–	–	–	8–190	–	Mas et al. (2020)
	Bandera	surface water	2014–2017	–	–	–	43	–	
Canada	Grand River	DWTP (raw water)	2015	ND (LOD = 3 ng/l)	2.7	77.1–138.1	13.5	18.2–42.9	Sultana et al. (2018)
	Lake Erie	DWTP (raw water)	2015	ND (LOD = 3 ng/l)	ND (LOD = 1 ng/l)	5.9–7.2	2.7–4.3	32.2–38.9	
	Detroit River	DWTP (raw water)	2015	ND (LOD = 3 ng/l)	ND (LOD = 1 ng/l)	6.8–33.2	4.4	52.7	
	Lake St. Clair	DWTP (raw water)	2015	ND (LOD = 3 ng/l)	ND (LOD = 1 ng/l)	28.7–86.9	3.7–8.6	10.2–283.5	
	Nicomekl River	surface water	2020	–	–	13–18	10–662	5	Manojlovic et al. (2021)
	Nicomekl River	surface water	2018	–	–	5–31.2	9.4–3 400	4.6–146	
	Nicomekl River	surface water	2017	–	–	5.6 –163	25–213	9.7–187	
USA	Minnesota	rivers and streams	2019	ND – 1.5 (LOD = 0.42 ng/l)	–	ND – 38 (LOD = 0.42 ng/l)	ND – 11 (LOD = 0.23 ng/l)	ND – 8 (LOD = 0.12 ng/l)	Berens et al. (2021)
	Minnesota	lakes	2019	ND (LOD = 0.42 ng/l)		ND – 1.6 (LOD = 0.42 ng/l)	ND – 3.6 (LOD = 0.23 ng/l)	ND – 1.4 (LOD = 0.12 ng/l)	
	Iowa City	tap water	2016	–	–	3.89–33.46	1.22–26.36	0.26–4.15	Klarich et al. (2017)
	Iowa	wells (raw drinking water)	2017–2018	ND	ND	< 0.05–13.4	< 0.09–2.4	< 0.03–20.6	Thompson et al. (2021)
China	Taihu Lake	surface water	2018	0.87–8.73	–	–	7.24–65.8	1.24–10	Zhou et al. (2020)
	Shanghai	DWTP (raw water)	2018–2019	10.35	–	–	21.26	13.19	Dong et al. (2021)
	Shanghai	DWTP (treated water)	2018–2019	5.49	–	–	10.97	9.57	
	Huangpu River	surface water	2018–2019	2.3–44.30	–	–	4–170.2	1.10–156.7	Xu et al (2020)
	Yangtze River Delta	river water	2016	2.213–58.487	–	–	10.924–1 886.882	2.974–90.848	Peng et al. (2018)
	Qing Reservoir – Yangtze River	DWTP (raw water)	2016	1.86	–	–	2.48	6.69	Troger et al. (2021)
	Jin Reservoir – Huangpu River	DWTP (raw water)	2016	8.21	–	–	6.32	4.75	
	Hainan	surface water	2018–2019	0–3 420	–	–	0–8 630	–	Tan et al. (2021)
Indonesia	Indramayu Regency	estuarine water	2020	–	1.77	–	8.75	7.13	Putri et al. (2022)
Japan	Surface water	DWTP (raw water)	2016	1.08	–	–	1.29	3.23	Troger et al. (2021)
Saudi Arabia	Al-Hassa Oasis	surface water	2017–2018	0–12.2	–	–	0–445	0–10.8	Pico et al. (2020)
Vietnam	Hanoi	lake water	2019	5.37	–	–	1.93	0.81	Wan et al. (2021)
	Hanoi	river water	2019	0.25	–	–	0.33	0.23	
	Hanoi	tap water	2019	0.07	–	–	0.06	0.19
	Saigon River	DWTP (raw water)	2016	7.59	–	–	5.18	9.18	Troger et al. (2021)

DWTP = drinking water treatment plant; LOD = limit of detection; ND = not detected

*Data obtained from Mgr. Vít Kodeš, Ph.D., head of Water Quality section of the Czech Hydrometeorological Institute

### Effects of selected neonicotinoids to aquatic organisms

Neonicotinoids can have significant sublethal and lethal effects on many aquatic invertebrates (Morrissey et al. 2015; Pagano et al. 2020). Aquatic invertebrates are a crucial component of ecosystems and form an essential link for energy flow between trophic layers. Invertebrates are important predators, parasites, and decomposers; they form the food base for many organisms from higher levels of the food chain (Covich et al. 1999). For their susceptibility to water contamination, invertebrates are excellent bioindicators for evaluating the presence of pollutants and the state of the ecosystem (Borges et al. 2021).

Acute and chronic toxicity of neonicotinoid insecticides significantly vary between species; the most sensitive orders are mayflies (Ephemeroptera), caddisflies (Trichoptera) and some species of Diptera, especially larvae of some midges (*Chironomidae*). Some species of these orders of insects already show a lethal effect at concentrations below 1 μg/l (Morrissey et al. 2015). With an increased exposure time, the LC50 (concentration that causes the death of 50% of tested organisms) value decreases (Sanchez-Bayo and Tennekes 2020).

Until it was banned, thiacloprid was one of the most widely used pesticide substances in the EU. Currently, acetamiprid and flupyradifurone are the only authorised substances for outdoor use in the EU. There are a relatively large number of studies on the toxic effects of acetamiprid and thiacloprid on aquatic organisms. However, there are few studies on the effects of flupyradifurone. Most of the available studies deal with the effects of the active substance, but only a few studies deal with the effects of the pesticide product itself. The basic characteristics of thiacloprid (THA), acetamiprid (ACE) and flupyradifurone (FLU) are presented in Table 3. The acute toxicity of THA, ACE and FLU for the selected aquatic organisms is presented in Table 4. The chronic toxicity of the same solutions for the selected aquatic organisms is presented in Table 5. Acute and chronic exposure to neonicotinoids has been shown to affect a range of aquatic organisms. During acute exposure, the larvae and adults of mosquitoes, freshwater amphipods, mayflies and other invertebrates appear to be the most sensitive. Lesser effects were then observed on Bivalvia, fish and amphibians. The chronic exposure of invertebrates usually affects the hatching, larval development, and mortality. Altered feeding strategies have also been observed. The chronic exposure of fish usually affects the hatching, development, growth, reproduction, enzymatic antioxidants biomarkers and oxidative stress. However, a shortcoming of many studies is the unclear methodology and the use of concentrations that are unrealistic to occur in the environment.

**Table 3:** Basic thiacloprid, acetamiprid and flupyradifurone characteristics

Characteristic	Thiacloprid	Acetamiprid	Flupyradifurone
Chemical name	[3-[(6-chloropyridin-3-yl)methyl]-1,3-thiazolidin-2-ylidene]cyanamide	*N*-[(6-chloropyridin-3-yl)methyl]-*N*'-cyano-*N*-methylethanimidamide	3-[(6-chloropyridin-3-yl)methyl-(2,2-difluoroethyl)amino]-2*H*-furan-5-one
Molecular formula	C_10_H_9_ClN_4_S	C_10_H_11_ClN_4_	C_12_H_11_ClF_2_N_2_O_2_
CAS	111988-49-9	160430-64-8	951659-40-8
Molecular weight (g/mol)	252.72	222.67	288.68
Colour	yellowish	white	–
Form	crystalline powder	crystals, crystalline solid	–
Odour	odourless	odourless	–
Solubility in water (g/l)	0.185	4.2	–
Soluble in	water, dichloromethane, *n*-octanol, *n*-propanol, acetone, ethyl acetate, polyethylene glycol, acetonitrile, DMSO	water, acetone, methanol, ethanol, dichloromethane, chloroform, acetonitrile, tetrahydrofuran	–
log K_ow_	1.26 at 20 °C	0.80 at 25 °C	–
Date of approval in EU	01.01.2005	01.01.2005	09.12.2015
Expiration of approval in EU	03.02.2020	28.02.2033	09.12.2025
Chemical structure depiction	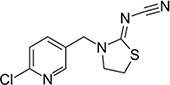	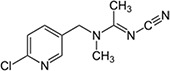	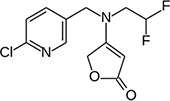
References	PubChem (2023a)	PubChem (2023b)	PubChem (2023c)

**Table 4:** Acute toxicity of acetamiprid, flupyradifurone and thiacloprid for selected aquatic organisms

Type of organism	Common name	Scientific name	Pesticide	Age/size	Endpoint	Toxicity (mg/l)	Other effects	References
Crustacea	Water flea	*Daphnia magna*	acetamiprid	< 24 hours	48hEC50	50	–	EPA (2023)
			flupyradifurone	< 24 hours	48hEC50	> 77.6	–	
			thiacloprid	< 24 hours	48hEC50	22.52	–
		*Ceriodaphnia dubia*	acetamiprid	< 24 hours	48hLC50	> 33.5	–	Raby et al. (2018)
			thiacloprid	< 24 hours	48hLC50	> 41.5	–	
	Mysid	*Americamysis bahia*	acetamiprid	< 24 hours	96hLC50	0.066	–	EPA (2023)
			flupyradifurone	< 24 hours	96hLC50	0.25	–	
			thiacloprid	< 24 hours	96hLC50	0.031	–	
	Freshwater amphipod	*Hyalella azteca*	acetamiprid	2–10 days	96hLC50	0.004 7	–	Bartlett et al. (2019)
			acetamiprid	2–9 days	96hLC50	0.004 8	–	Raby et al. (2018)
			flupyradifurone	2–10 days	96hLC50	0.026	–	Bartlett et al. (2019)
			thiacloprid	2–10 days	96hLC50	0.068	–	
			thiacloprid	14–21 days	96hLC50	0.037	–	EPA (2023)
			thiacloprid	2–9 days	96hLC50	0.363 2	–	Raby et al. (2018)
	Black Tiger shrimp	*Penaeus monodon*	acetamiprid	67–70 days	48hLC50	> 0.500	↑CAT, GST, AChE	Butcherine et al. (2021)
	Isopod	*Caecidotea* sp.	acetamiprid	adults	96hLC50	2.129 6	–	Raby et al. (2018)
	Common yabby	*Cherax destructor*	Calypso 480 SC (thiacloprid 480 g/l)	7.04 ± 3.4 g	96hLC50	7.7	movement decreased with increasing concentration; behavioural changes in conc. from 5 mg/l; ↓LPO	Stara et al. (2019)
	Scud	*Gammarus asciatus*	acetamiprid	N.R.	96hEC50	0.08	–	EPA (2023)
Worm	California blackworm	*Lumbriculus variegatus*	acetamiprid	7 days	96hLC50	0.026 5	–	Raby et al. (2018)
			thiacloprid	7 days	96hLC50	0.033 8	–	
Insects	Midge	*Chironomus riparius*	acetamiprid	4 days	48hLC50	0.209	–	EPA (2023)
			flupyradifurone	< 3 days	48hLC50	0.063 9	–	
		*Chironomus dilutus*	acetamiprid	3^rd^ instar	96hLC50	0.002 8	–	Raby et al. (2018)
			flupyradifurone	larvae	96hLC50	0.016 6	–	Maloney et al. (2020)
			thiacloprid	3^rd^ instar	96hLC50	0.001 6		Raby et al. (2018)
	Eurasian Bluet	*Coenagrion* sp.	acetamiprid	nymphs	96hLC50	24.392 9	–	Raby et al. (2018)
			thiacloprid	nymphs	96hLC50	5.647 2	–	
	Water boatmen	*Trichocorixa* sp.	acetamiprid	adults	48hLC50	1.515 2	–	Raby et al. (2018)
			thiacloprid	adults	48hLC50	0.135 3	–	
	Caddisfly	*Cheumatopsyche* sp.	acetamiprid	nymphs	96hLC50	0.403 8	–	Raby et al. (2018)
			thiacloprid	nymphs	96hLC50	> 0.92	–	
	Whirligig beetle	*Gyrinus* sp.	acetamiprid	adults	96hLC50	0.686 5	–	Raby et al. (2018)
			thiacloprid	adults	96hLC50	0.180 9	–	
	Riffle beetle	*Stenelmis* sp.	acetamiprid	adults	96hLC50	0.238 3	–	Raby et al. (2018)
			thiacloprid	adults	96hLC50	0.183 6	–	
	Mosquito	*Culex quinquefasciatus*	acetamiprid	adults	48hLC50	0.000 56	–	Shah et al. (2016)
			Mospilan 20 SP (acetamiprid 20%)	larvae	48hLC50	0.000 005–0.000 104	–	Kamran et al. (2022)
		*Culex pipiens*	Acetivot 20% WP (acetamiprid 20%)	larvae	72hLC50	0.006 5	↑AChE; GST	Abdel-Haleem et al. (2020)
		*Aedes* sp.	acetamiprid	larvae	48hLC50	0.159 6	–	Raby et al. (2018)
			thiacloprid	larvae	48hLC50	0.053 4	–	
	Mayfly	*Ephemerella* sp.	acetamiprid	nymphs	96hLC50	0.158 2	–	Raby et al. (2018)
			thiacloprid	nymphs	96hLC50	0.190 6	–	
		*Hexagenia* spp.	acetamiprid	nymphs	96hLC50	0.78	–	Bartlett et al. (2018)
			acetamiprid	4–6 mg	96hLC50	> 35.6	–	Raby et al. (2018)
			flupyradifurone	nymphs	96hLC50	2	–	Bartlett et al. (2018)
			thiacloprid	nymphs	96hLC50	6.2	–	
			thiacloprid	4–6 mg	96hLC50	> 9.3	–	Raby et al. (2018)
		*Isonychia bicolor*	acetamiprid	nymphs	96hLC50	> 9.6	–	Raby et al. (2018)
		*McCaffertium* sp.	acetamiprid	nymphs	96hLC50	> 0.89	–	Raby et al. (2018)
			thiacloprid	nymphs	96hLC50	0.92	–	
		*Cloeon* sp.	acetamiprid	nymphs	96hLC50	2.369 7	–	Raby et al. (2018)
			thiacloprid	nymphs	96hLC50	3.826	–	
		*Neocleon triangulifer*	acetamiprid	< 24 hours	96hLC50	0.001 7	–	Raby et al. (2018)
			thiacloprid	< 24 hours	96hLC50	0.019	–	
		*Caenis* sp.	acetamiprid	nymphs	96hLC50	0.782 8	–	Raby et al. (2018)
			thiacloprid	nymphs	96hLC50	0.231 4	–	
Bivalvia	Mediterranean mussel	*Mytilus galloprovincialis*	thiacloprid	6.85 ± 0.57 cm	96hLC50	> 10	↑CAT in gills after 3 days of exposure to 10 mg/l; ↓CAT in digestive gland after 7 days of exposure to 5 mg/l	Stara et al. (2020a)
			Calypso 480 SC (thiacloprid 480 g/l)	6.85 ± 0.57 cm	96hLC50	> 100	↓CAT in digestive gland after 3 days of exposure to 100 mg/l and in gills after 10 days in all concentrations; ↓SOD in gills after 3 days in all concentrations	
	Eastern oyster	*Crassostrea virginica*	acetamiprid	spat	96hLC50	41	–	EPA (2023)
			flupyradifurone	spat	96hLC50	> 29	–	
			thiacloprid	spat	96hLC50	4	–	
Fish	African catfish	*Clarias gariepinus*	acetamiprid	juveniles	96hLC50	265.7	–	Houndji et al. (2020)
	Nile tilapia	*Oreochromis niloticus*	Telfast 20 SP (acetamiprid 20%)	juveniles	96hLC50	195.813	–	El-Garawani et al. (2022)
			Telfast 20 SP (acetamiprid 20%)	juveniles	96hLC50	202.35	–	Hathout et al. (2021)
	Rainbow trout	*Oncorhynchus mykiss*	acetamiprid	2.05 g	96hLC50	> 100	–	EPA (2023)
			flupyradifurone	0.79 g	96hLC50	> 74.2	–	
			thiacloprid	1.2 g	96hLC50	30.2	–	
	Eastern mosquitofish	*Gambusia holbrooki*	RastT 20SP (acetamiprid 20%)	3.5 ± 0.07 cm; 0.54 ± 0.16 g	96hLC50	42.2	significant changes in GST; GR	Demirci and Gungordu (2020)
	Major South Asian carp	*Catla catla*	acetamiprid	10–15 g	96hLC50	–	↓CAT, SOD, GST, GSH in gill; ↓LPO increase	Veedu et al. (2022)
	Grass carp	*Ctenopharyngodon idela*	Telfast 20 SP (acetamiprid 20%)	30 ± 2 g	96hLC50	121.146	–	Azadikhah et al. (2023)
	Zebrafish	*Danio rerio*	acetamiprid	larvae (5 dpf)	96hLC50	58.39	–	Hu et al. (2023)
			acetamiprid	embryo	96hLC50	143.9	–	
			acetamiprid	adults	96hLC50	10.36	↑GST in brain and liver	Wang et al. (2018b)
			acetamiprid	juvenile	96hLC50	36.91	–	
			acetamiprid	larvae	96hLC50	15.52	–	
			acetamiprid	embryo	96hLC50	13.33	–	
			flupyradifurone	5.5 hpf	96hLC50	210	↓heart rate, body length, survival rate; abnormalities in cardiac development (elongated pericardium, pericardial edema aggravation, increased atrial ventricular spacing, increased degree of the un-looped heart; ↓CAT, SOD	Zhong et al. (2021)
	Fathead minnow	*Pimephales promelas*	flupyradifurone	0.85 g	96hLC50	> 70.5	–	EPA (2023)
			thiacloprid	0.24	96hLC50	> 104	–	
	Common carp	*Cyprinus carpio*	flupyradifurone	1.7 g	96hLC50	> 80	–	EPA (2023)
	Sheepshead minnow	*Cyprinodon variegatus*	acetamiprid	0.53 g	96hLC50	100	–	EPA (2023)
			flupyradifurone	0.24 g	96hLC50	> 83.9	–	
			thiacloprid	0.23 g	96hLC50	19.7	–	
Amphibians	Western clawed frog	*Silurana tropicalis*	acetamiprid	tadpole	96hLC50	> 100	–	Saka and Tada (2021)
	African clawed frog	*Xenopus laevis*	acetamiprid	tadpole	96hLC50	64.48	–	Jiao et al. (2023)
			Calypso OD240 (thiacloprid 240 g/l)	tadpole	96hLC50	13.41	–	Uckun and Ozmen (2021)
	Dark-spotted frog	*Rana nigromaculata*	acetamiprid	tadpole	LC50	18.49	–	Guo et al. (2022)

48hEC50 = concentration causing inhibition of 50 % of test organisms in 48 hours; 48hLC50 = concentration causing mortality of 50 % of test organisms in 48 hours; 96hLC50 = concentration causing mortality of 50 % of test organisms in 96 hours; AChE = enzymatic activity of acetylcholine esterase; CAT = enzymatic activity of catalase; dpf = days post fertilisation; GR = enzymatic activity of glutathione reductase; GSH = concentration of glutathione; GST = enzymatic activity of glutathione-*S*-transferases; hpf = hours post fertilisation; LPO = lipid peroxidation; SOD = enzymatic activity of superoxide dismutasew

**Table 5:** Chronic toxicity of acetamiprid, flupyradifurone and thiacloprid for selected aquatic organisms

Type of organism	Common name	Scientific name	Pesticide	Study length	Used concentrations	LOEC (mg/l)	NOEL (mg/l)	Other effects	References
Crustaceans	Water flea	*Daphnia magna*	flupyradifurone	21 days	–	6.73	3.42	–	EPA (2023)
			acetamiprid	21 days	–	9	5	–	
			thiacloprid	21 days	–	1.01	0.56	–	
	Marine copepod	*Acartia tonsa*	thiacloprid	26 days (21 days for F0 + 5 days for F1)	10 and 100 ng/l	–	–	hatching affected; larvae development inhibited	Picone et al. (2022)
			acetamiprid	26 days (21 days for F0 + 5 days for F1)	10 and 100 ng/l	–	–	↓egg production; hatching affected; larvae development inhibited; ↑larval mortality	
	Freshwater amphipod	*Gammarus fossarum*	Calypso 480 SC (thiacloprid 480 g/l)	7 days	0.75–6 μg/l	–	–	↓leaf consumption; ↑predation on Baetis nymphs	Bundschuh et al. (2020)
	Mysid	*Americamysis bahia*	flupyradifurone	28 days	–	23.6	1.32	–	EPA (2023)
			acetamiprid	28 days	–	0.004 7	0.002 5	–	
			thiacloprid	32 days	–	0.002 2	0.001 1	–	
Insects	Midge	*Chironomus riparius*	flupyradifurone	28 days	–	0.021 3	0.010 5	–	EPA (2023)
			acetamiprid	28 days	–	0.01	0.005	–	
			thiacloprid	28 days	–	0.003 2	0.001 8	–	
Gastropods	Mediterranean mussel	*Mytilus galloprovincialis*	thiacloprid	7 days	4.5 and 450 μg/l	–	–	histological damage to the digestive gland and gills; ↓CAT; GST; LPO	Stara et al. (2021)
			Calypso 480 SC (thiacloprid 480 g/l)	20 days; 10 days recovery period	7.77 and 77.7 mg/l	–	–	↓haemolymph parameters (Cl^-^, Na^+^); affected SOD of digestive gland and CAT of gill; histopathological alterations in digestive gland and gills	Stara et al. (2020b)
Fish	Common carp	*Cyprinus carpio*	thiacloprid	35 days	4.5; 45; 225; 450 μg/l	–	–	↓lower weight and length; ↓SOD and GR activity	Velisek and Stara (2018)
	Zebrafish	*Danio rerio*	acetamiprid	154 days	0.19–1 637 μg/l	–	–	feminization and reproductive dysfunction in zebrafish; impaired production and development of offspring	Ma et al. (2022)
	Nile tilapia	*Oreochromis niloticus* (juveniles)	Telfast 20 SP (acetamiprid 20%)	21 days	19.5 mg/l (representing 96hLC50/10)	–	–	colour darkening; sluggish swimming; raised fins; lethargy; enlarged dark gall bladders	El-Garawani et al. (2022)
			Telfast 20 SP (acetamiprid 20%)	21 days	10; 20 mg/l	–	–	↓SOD, GPx; production of LPO substances in fish liver	Hathout et al. (2021)
	Rainbow trout	*Oncorhynchus mykiss* (early lyfestages)	thiacloprid	97 days	–	1.91	0.92	–	EPA (2023)
	Fathead minnow	*Pimephales promelas*	flupyradifurone	35 days	–	8.4	4.4	–	EPA (2023)
			acetamiprid	35 days	–	38.4	19.2	–	
			thiacloprid	33 days	–	> 0.170	0.17	–	
				106 days	–	> 0.710	0.71	–	
				260 days	–	–	–	–	
Amphibians	African clawed frog	*Xenopus laevis* (tadpole)	acetamiprid	28 days	0.645 and 6.45 mg/l (representing 1/100 and 1/10 96hLC50)	–	–	↑melano-macrophages; obscure liver cords; inflammatory infiltration in liver tissues	Jiao et al. (2023)
		*Rana nigromaculata* (tadpole)	acetamiprid	28 days	0.185 and 1.85 mg/l	–	–	↑CAT, SOD, GR, GST ↓AChE	Guo et al. (2022)
	Egyptian toads	*Sclerophrys regularis* (adults)	Acetamore 20% (acetamiprid 20%)	14 days	40 mg/l	–	–	↑the serum levels of total lipid, cholesterol, triglyceride, AST, ALT; ↓in hepatic GSH and SOD; ↑MDA	Saad et al. (2022)
	Western clawed frog	*Silurana tropicalis* (tadpole)	acetamiprid	26–28 days	0.1 and 1 mg/l (representing 1/10 and 1/100 of 96hLC50)	–	–	no significant differences in any of the endpoints (mortality, malformations and other visually recognisable abnormalities)	Saka and Tada (2021)

AChE = enzymatic activity of acetylcholine esterase; ALT = alanine aminotransferase; AST = aspartate aminotransferase; CAT = enzymatic activity of catalase; dpf = days post fertilisation; GPx = enzymatic activity of glutathione peroxidase; GR = enzymatic activity of glutathione reductase; GSH = concentration of glutathione; GST = enzymatic activity of glutathione-*S*-transferases; hpf = hours post fertilisation; LOEC = lowest observed effect concentration; LPO = lipid peroxidation; MDA = malondialdehyde; NOEC = no observed effect concentration; SOD = enzymatic activity of superoxide dismutase

The initiation of downstream drift may be a sublethal effect of neonicotinoids, especially in running water organisms (Beketov and Liess 2008). Another observed phenomenon of organisms during exposure to neonicotinoids is a reduced ability to eat, even after being relocated to a clean environment (Alexander et al. 2007). When evaluating neonicotinoids and other substance effects, not only the lethal and sublethal effects to organisms should be evaluated, but also community-wide effects, the interactions between the organisms and the functionality of the whole ecosystem should also be addressed (Hladik et al. 2018). The individual components of ecosystems are closely interconnected, although neonicotinoids do not cause vertebrate mortality directly, they act on them through their food base. The reduction in invertebrate abundance correlates with the reduction in the abundance of animals whose food base consists mainly of invertebrates (Sanchez-Bayo et al. 2016). As stated by Hayasaka et al. (2012), the recovery of populations affected by neonicotinoids is very challenging and slow, so it can be assumed that the return of aquatic invertebrate predators will also be slow. One of the basic functions of ecosystems is the decomposition of organic matter, which, among others, the larvae of mayflies (Ephemeroptera), caddisflies (Trichoptera) and stoneflies (Plecoptera), are also sensitive, which are also considered as bioindicators of water quality (Morse et al. 1993). If these organisms are reduced by neonicotinoids, a reduction in their deterrent activity also occurs. This phenomenon can also materialise as a sublethal effect (Kreutzweiser et al. 2008; Bundschuh et al. 2020). The decomposition of organic matter affects the water quality in its recipients. The deterioration in the water quality can, thus, be one of the indicators of the presence of pollutants in the environment (Sanchez-Bayo et al. 2016).

### Neonicotinoids in the Czech Republic

**Figure 1:**
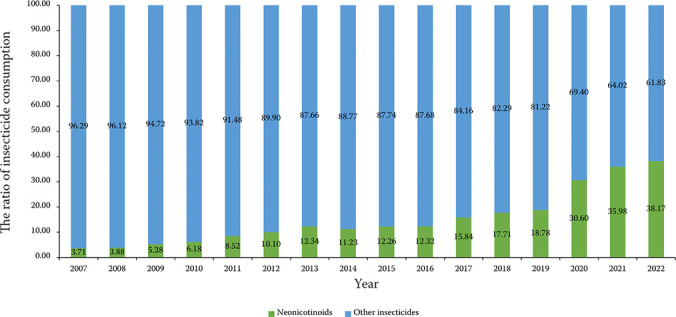
The ratio of neonicotinoid consumption to insecticide consumption in the Czech Republic (in %) (CISTA 2023)

**Figure 2:**
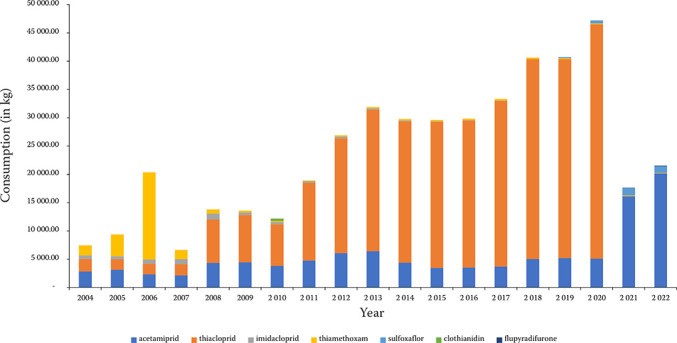
The trend in consumption of neonicotinoid substances registered in the Czech Republic (in kg) (CISTA 2023)

The success and use of neonicotinoids in agriculture can be demonstrated by their usage in the Czech Republic. In 2007, they accounted for less than 4% of the total usage of insecticides in the Czech Republic. Even though the total consumption of insecticides in the Czech Republic has decreased since 2018, the share of neonicotinoids in the consumption is on the contrary increasing. While it was less than 18% in 2018 and less than 19% in 2019, from 2020, the neonicotinoid consumption covers 1/3 of the total insecticide consumption in the Czech Republic. The ratio of neonicotinoid consumption to insecticide consumption in the Czech Republic is shown in Figure 1. Since the beginning of neonicotinoid use, acetamiprid and thiacloprid have been the most used neonicotinoid substances, followed by imidacloprid and thiamethoxam to a lesser extent. The changing EU legislation and gradual bans of the selected substances are highly evident in the trends of neonicotinoid use. The trend in consumption of individual neonicotinoid substances registered in the Czech Republic is shown in Figure 2. Up to 85% of the neonicotinoids consumed in the Czech Republic are applied to oilseeds and around 10% are applied to cereals (CISTA 2023).

## CONCLUSION

As one of the most progressive groups of insecticides, neonicotinoids are also one of the most detected pesticides in global waters. Their success and popularity can be demonstrated by the example of the Czech Republic, where they currently occupy more than 1/3 of the total insecticide market. Although they appeared to be of low toxicity to non-target organisms and invertebrates in general when they were introduced, several studies have shown that these claims are not entirely true. A number of neonicotinoids are highly toxic to pollinators and, for this reason, the EU has taken measures to restrict the use and even ban certain neonicotinoids altogether within the EU. Acute and chronic exposure to neonicotinoids has been shown to affect a range of aquatic organisms. During acute exposure, the larvae and adults of mosquitoes, freshwater amphipods, mayflies and other invertebrates appear to be most sensitive. Lesser effects were then observed on Bivalvia, fish and amphibians. The chronic exposure of invertebrates usually affects the hatching, larval development, and mortality. Altered feeding strategies have also been observed. The chronic exposure of fish usually affects the hatching, development, growth, reproduction, enzymatic antioxidants biomarkers and oxidative stress. However, a shortcoming of many studies is the unclear methodology and the use of concentrations that are unrealistic to occur in the environment. However, threats to individual species of organisms can pose a problem for their entire populations, even for entire ecosystems.
